# Traumatic brain injury: molecular biomarkers, genetics, secondary consequences, and medical management

**DOI:** 10.3389/fnins.2024.1446076

**Published:** 2024-10-04

**Authors:** Robert H. Lipsky, Jeffrey M. Witkin, Hana Shafique, Jodi L. Smith, Rok Cerne, Ann M. Marini

**Affiliations:** ^1^Department of Neurology, Uniformed Services University of the Health Sciences, Bethesda, MD, United States; ^2^Program in Neuroscience, and Molecular and Cellular Biology Program, Uniformed Services University of the Health Sciences, Bethesda, MD, United States; ^3^Laboratory of Antiepileptic Drug Discovery Ascension St. Vincent Hospital, Indianapolis, IN, United States; ^4^Departments of Neuroscience and Trauma Research Ascension St. Vincent Hospital, Indianapolis, IN, United States; ^5^Duke University School of Medicine, Durham, NC, United States

**Keywords:** molecular biomarkers, genetics, secondary consequences, and medical management traumatic brain injury, genes, neurodegenerative disorders, biomarkers, inflammation

## Abstract

Traumatic brain injury (TBI) has reached epidemic proportions worldwide. The consequences of TBI can be severe even with repetitive mild trauma. If death and coma are avoided, the consequences of TBI in the long term typically involve dizziness, sleep disturbances, headache, seizures, cognitive impairment, focal deficits, depression, and anxiety. The severity of brain injury is a significant predictor of outcome. However, the heterogenous nature of the injury makes prognosis difficult. The present review of the literature focuses on the genetics of TBI including genome wide (GWAS) data and candidate gene associations, among them brain-derived neurotrophic factor (BDNF) with TBI and development of post-traumatic epilepsy (PTE). Molecular biomarkers of TBI are also discussed with a focus on proteins and the inflammatory protein IL1-β. The secondary medical sequela to TBI of cognitive impairment, PTE, headache and risk for neurodegenerative disorders is also discussed. This overview of TBI concludes with a review and discussion of the medical management of TBI and the medicines used for and being developed at the preclinical and clinical stages for the treatment of TBI and its host of life-debilitating symptoms.

## 1 Introduction

Since the first report on traumatic brain injury (TBI) in 1891, there have been major efforts to scientifically explore this highly prevalent condition. TBI has serious devasting effects on the quality of life of patients, their caregivers, drains the finances of families, and negatively impacts the economy through lost work productivity. TBI results in major medical issues post trauma that include cognitive impairment, focal deficits, headache, pain, seizures, behavior disorders, and potentially neurodegenerative disorders. There are no medications that are specific for TBI patients such that the medical course is symptom management with the additional hope of slowing the progression of secondary effects of injury.

Traumatic brain injury is a common around the world and a leading cause of morbidity and mortality among children, young adults, and the elderly in the United States. In children and elderly, falls are a major concern, while in young adults, motor vehicle accidents are a major contributor. Fortunately, there has been a 20% reduction in the incidence of TBI in the United States over the past 20 years, largely due improved automobile safety standards. Still, an estimated 1.5 million Americans sustain a TBI every year. Recent data show that 611 TBI-related hospitalizations and 176 TBI-related deaths occur every day. Accordingly, 5.3 million Americans have a TBI-related disability. Older adults, adolescents and young adults are at the highest risk of sustaining a TBI. The financial cost is estimated in the billions per year for those with TBI-induced long-term disabilities, but the physical care and emotional distress imposed on family members are incalculable (Centers for Disease Control and Prevention [CDC], 2024).

There are two types of TBI, closed head and penetrating injury. Examples of closed head TBIs are: motor vehicle accidents, falls, and violence whereas an example of penetrating trauma is a gunshot wound to the head. Approximately, 64,000 Americans died from a TBI in 2020. Men were more likely to be hospitalized and die from a TBI than women. TBI-related suicide rates reached 35.5% in the United States from 2018 to 2019; adults older than 75 years had the highest average annual rate of suicide. The highest rate of TBI-related deaths occurred in Alaskan, American Indian and non-Hispanic individuals between 2018 and 2019. The TBI-related suicide rate in those from age 15 to 74 ranged from 6.5 to 8.7 per 100,000 in 2019. The most common injuries are due to falls, motor vehicle accidents, and injuries sustained while walking along a street, riding a bicycle or motorcycle (Centers for Disease Control and Prevention [CDC], 2018/2019). These results indicate that a broad approach should be undertaken between healthcare providers and the community to educate and instruct the public about common causes of TBI and suicide.

The search continues for new experimental treatments. As of 04 October 2023, there were 66,546 papers listed under the topic in https://pubmed.ncbi.nlm.nih.gov/. This is a small percentage of research reports when compared to other areas of disease (e.g., 1.3% of cancer; 2.3% of cardiovascular disease; 36.9% of epilepsy; 40.6% of schizophrenia papers) but more than other disease areas (e.g., 889% of Fragile X syndrome; 152% of Down syndrome). We suggest that the relatively small research investment in TBI is due to the lack of firm traction that has been gained in therapeutic interventions. For example, research in the area of other traumas where life support and symptom management are generally the medical model are, despite their high prevalence like TBI, are also represented less than other disorders in the scientific domain; the amount of research on blunt trauma has been about equivalent to that of TBI with nearly equivalent research durations of about a century.

## 2 Injury severity

Traumatic brain injury is the leading cause of disability among all traumatic injuries (Dewan et al., 2018). Traumatic brain injury is categorized according to severity and ranges from mild to severe. The new definition of mild TBI (mTBI) by the American Congress of Rehabilitation Medicine (Silverberg et al., 2023) is “diagnosed when, following a biomechanically plausible mechanism of injury (Criterion 1) one or more of the criteria (i-iii)” listed are met: i) one or more clinical signs (Criterion 2) of TBI; ii. At least two acute symptoms (Criterion 3) and at least one clinical or laboratory finding (Criterion 4) due to TBI; iii. Neuroimaging evidence of TBI such as trauma-related intracranial lesions on structural brain MRI (Criterion 5). A loss of consciousness for more than 30 min, a GCS score of less than 13 after 30 minutes and/or post-traumatic amnesia for more than 24 hours are signs and/or symptoms that are inconsistent with a diagnosis of mTBI. If there is a lesion on brain MRI, along with other criteria consistent with an mTBI, one can use the phrase mTBI with neuroimaging evidence of an “structural intracranial injury.” Criterion 1 can be made based upon a direct observation, witness, review of the medical record or personal history. Criterion 2 (Clinical Signs) is the observed clinical signs including loss of consciousness immediately following the injury, alternation of mental status immediately following the injury as a loss of awareness/consciousness for 30 min or less, a Glasgow coma scale (GCS) of 13–15 after 30 min and post-traumatic amnesia for less than 24 hours. A moderate TBI is defined as a loss of awareness/consciousness of greater than 30 min to less than 24 hours, a GCS of 9–12 and post-traumatic amnesia of 1–7 days and a severe TBI is defined as a GCS of 3–8, post-traumatic amnesia of longer than 7 days and loss of consciousness of greater than 24 hours. The greater the magnitude of injury the greater the damage to the brain and the worse the outcome. In addition to mild and moderate classifications, there is a special exception to categorizing mTBI, called mild complicated. Mild complicated or mTBI with evidence of an intracranial injury on head CT has a higher symptom burden and poorer functional outcomes (Voormolen et al., 2020; Howe et al., 2022). Etiologies excluded from these definitions include alcohol, medications, facial or systemic injuries, penetrating injuries to the brain, infection or coexisting medical conditions.

## 3 Genetics

Inquiry into the genetic associations with different aspects of vulnerability, injury severity, and recovery following TBI allows further understanding of specificpathophysiological mechanisms. This approach alsocreates opportunities for discovery of potential biomarkers, and may inform new treatment options (see Discussion). Due to statistical considerations, the majority of studies focus on “candidate gene” associations with phenotypes in numerically small patient populations. However, more recent genome-wide association studies (GWAS) offer the opportunity to better describe a condition where the phenotype(s) is modifed by multiple genes.

### 3.1 Genome-wide association study (GWAS) findings of genetic associations with TBI susceptibility

Genetic factors are known to modify the response to injury and are thought to involve multiple loci. The results of a recent multi-site GWAS performed in military veterans (Merritt et al., 2024) reinforced this idea of polygenicity and revealed stronger associations with behavioral phenotypes such as risk-taking rather than with psychiatric traits. Analysis identified 15 genome-wide significant loci at a significance level of *p* < 5x10^–8^. Gene-based analyses revealed 14 gene-wide significant genes, including *NCAM1*, *APOE*, *FTO*, and *FOXP2*. The genetic markers, single nucleotide polymorphisms (SNPs), showing positive association were confined to those individuals of European origin. No genome-wide significant genes were identified in veteran African Americans. That result may be due the lower numbers of African Americans represented in the veteran cohort having decreased statistical power to detect an association between genes and TBI risk. Gene expression analysis identified the brain as significantly enriched, particularly in the frontal cortex, anterior cingulate cortex, and nucleus accumbens. Genetic correlations with TBI were significant for risk taking behaviors and psychiatric disorders, but generally not significant for the neurocognitive variables analyzed in this study. A major strength of the experimental approach was the sample size (n = 304,485; 111,494 TBI cases, 192,991 controls), with separate replication cohorts (15,787 “concussion” cases, 184,565 no TBI controls) and one for “severe TBI” (4,927 cases, 304,227 no TBI controls). The goals of future TBI GWASs should be focused on increasing ethnic population sample sizes, injury severity and diversity, chronic post-concussive symptoms, as well as outcomes.

### 3.2 Genetic factors and recovery from TBI

The majority of genetic association studies conducted on TBI have used a candidate gene approach on small populations of TBI patients One genetic factor that has consistently had a role in TBI is a functional variation of apolipoprotein E (ApoE) and its influence in overall patient outcome and cognitive and behavioral functions following TBI. More than two decades of research has supported a role for genetic variation at the *APOE* gene with poorer outcome following TBI (Lipsky and Lin, 2015), specifically linking the E4 allele (*APOE4*) with poorer outcome. APOE influences rehabilitation outcome, including Functional Independence Measures (FIM) scores, coma recovery, risk of posttraumatic seizures, in addition to cognitive and behavioral performance following TBI (Kodaka et al., 2004). The first findings to show an association of the *APOE4* with poorer memory performance was reported in 2002 (Crawford et al., 2002). Functional alleles of the catechol *O*-methyltransferase gene (*COMT*) and the brain-derived neurotrophic factor gene (*BDNF*, below) were also linked with poorer cognitive performance. Although a number of other genes have been implicated in recovery from TBI, the candidate gene approach has only been partially successful in identifying and replicating functional genetic variants that are predictive of outcome (Lipsky and Lin, 2015).

### 3.3 Genetics of BDNF

BDNF is a member of the nerve growth factor family of secretory trophic factors with functions in CNS development and cell survival (Marini et al., 1998; Vajda, 2002). Within the propolypeptide region of the human *BDNF* gene there is a functional SNP changing a valine (Val) to methionine (Met) at codon 66 (rs6265) that reduces targeting of BDNF protein to secretory granules in the regulated pathway and reduces activity-dependent secretion by neurons maintained in the culture (Chen et al., 2004; Jiang et al., 2005; Jiang et al., 2009). Transgenic mice expressing human Met66 BDNF also have cognitive and behavioral defects similar to phenotypes seen in humans (Chen et al., 2006; Jiang et al., 2005; Yu et al., 2009). In human studies, the *BDNF* Met66 allele has been associated with memory impairments in healthy human subjects and in different disease cohorts. Kruger and colleagues tested the hypothesis that a cohort of Vietnam War veterans having penetrating brain injury to the prefrontal cortex would show improved executive functioning among Val66 carriers (Krueger et al., 2011). The results revealed that Met66 allele carriers, not the proposed Val allele, were associated with better executive functioning. An explanation for the divergent findings may be age-related effects of this SNP in this particular veteran population (average age of 59 years) and the influence of the *BDNF* Val66Met genotype on human hippocampal aging. In support of this concept, a functional magnetic resonance imaging study by Sambataro et al. (2010) showed that Met66 carriers had a significantly steeper slope in age-related decline in hippocampal activation during encoding and retrieval phases relative to Val/Val individuals, but Met66 carriers showed greater bilateral inferior frontal activation with increasing age during retrieval relative to Val/Val individuals. Thus, Met66 carriers may have a compensatory mechanism to maintain memory performance (Sambataro et al., 2010). A critical first step will be to replicate the findings in larger study populations.

### 3.4 Genetics of PTE

A common consequence of TBI is the development of PTE over time post trauma (See Section V below). Currently, there are no methods to prevent PTE. Genetic biomarkers may be useful to better understand etiology of the condition and to predict outcomes.

A study by Raymont et al. (2010) used a candidate gene approach based on the hypothesis that genetic variants in glutamate and GABA neurotransmitter pathways alters the balance between excitation and inhibition and that this predisposing condition may make individuals more susceptible to PTE following TBI. Vietnam War veterans were prospectively recruited, with longitudinal follow-up for up to 35 years following injury. The study originally recruited 1,221 individuals, mostly comprised of penetrating head injuries, and had a high prevalence (45%–53%) of PTE, that made it desirable to identify candidate risk genes for PTE. There were three phases for follow-up. Phase 1 (PH1) of the study was a review of subjects’ medical records 5 years after injury. Phase 2 (PH2) evaluated 520 subjects with head injuries from the original registry and 85 uninjured healthy volunteers. Phase (PH3) evaluated 199 veterans plus uninjured controls 30 to 35 years after injury. Of the 520 individuals assessed at PH2, 336 did not participate in PH3. When comparing the subjects with head injuries who participated in both PH2 and PH3, the prevalence of PTE at PH3 was increased to 45.1% (82 of 182 subjects), compared with 39.6% at PH2. 19.2% reported having a seizure in the year before PH3. As in the whole sample with PTE, in the most recent year of subject-reported seizures, the most common seizure frequency was two to 10 seizures per year, and complex partial seizures were the most common type, occurring in 15.1% of cases. Ninety-two percent of individuals had penetrating head injuries. Genetic analyses revealed that PTE was predicted by both the presence of the glutamate receptor subunit episilon 2A gene (*GRIN2A*) SNP rs11074504 and left parietal involvement (F = 5.931, df = 1, p = 0.041). None of these predictors remained significant when the analysis was repeated for very late-onset PTE only (14 years after injury). For participants at PH3, there was an association of the glutamate decarboxylase gene (*GAD2*) SNP, rs1330582, with PTE at PH2. There was a marginal association between *GAD1* gene SNP rs769395 and seizures at PH2. There was no association between PTE and the presence of the *APOE4*. Finally, when the significant p-values were corrected for multiple comparisons, none of the values remained significant. There was an association between the presence of *GRIN2A* SNP rs11074504 and seizures. However, because of the small numbers of subjects involved, this finding could only be considered as exploratory. The non-significant genetic findings underscored the challenges in recruiting sufficient numbers of participants that meet inclusion criteria and also emphasizes the importance of collecting phenotypic data that reflects underlying pathophysiology. Additionally, effect sizes of common (presumably functional) genetic variants are small, so designing adequately powered studies is critical to providing supportive evidence of a specific gene association with a disorder/phenotype or for confidence in excluding it. A recent review was published to test the association of SNPs with risk of PTE and post-stroke epilepsy (Misra et al., 2023). Although eleven SNPs were associated with significantly increased risk of PTE, no validation studies were published. A meta-analysis for an association of the *APOE 4* allele with PTE was nonsignificant. While multi-center studies for GWAS have been conducted for common epilepsies, no GWAS have been performed for PTE to date.

## 4 Molecular biomarkers of TBI

The heterogeneity and complexity of TBI will require the differentiation of pathological, clinical, demographic, genomic and proteomic underpinnings that affect treatment options, treatment response, and ultimately patient health outcomes. To date, there has been an inability to predict outcomes for TBI patients. To better stratify patients will require the collection of high-quality data in large cohorts of both patients and healthy controls. It is critical that improved diagnosis and prognosis be the first steps to demonstrating the benefit of new therapies; these improvements will also permit an identification of specific subsets of patients that are the best candidates for targeted individualized therapy. Improved stratification and categorization of brain injury, based on an improved detection of brain injury by advanced imaging, proteomic, and genetic testing will permit identification of more homogeneous subsets of patients to be targeted for specific types of therapy. Here, we briefly describe the progress in discovery and validation of protein biomarkers alone and in combination with genetic biomarkers to determineTBI risk and clinical outcomes following TBI.

### 4.1 Protein biomarkers of TBI

Necrotic cell death in the primary focus of TBI elicits a strong secondary inflammatory response particularly in microglia and astrocytes surrounding the site of injury (Otto et al., 2001). These cells respond to injury-induced free radicals by an increased expression and release of cytokines such as interleukin (IL) IL-1β, TNF-α, IL-6 and ciliary neurotrophic factor. Levels of the proinflammatory cytokine IL-6 was associated with the clinical course of injury (Gebhard et al., 2000). Other candidate biomarkers are known, among them is glial fibrillary acidic protein (GFAP), an astrocyte-specific cytoskeletal protein which is a robust biomarker associated with severe TBI (Wang et al., 2018). GFAP is not normally found in blood, but is detected following brain injury (Wang et al., 2018). S100B is an astroglial-associated protein that has shown some promise for predicting CT abnormality among mTBI patients, although it is not specific to CNS injury (Wang et al., 2018).

Tau proteins function to stabilize microtubules and are found in high levels in neurons. In particular phosphorylated isoforms result in the creation of tangles and are associated with Alzheimer’s disease and other “tauopathies” such as CTE. Elevated total tau protein levels have been shown to be in serum of patients with severe TBI at least six hours following injury and in plasma of Olympic (amateur) boxers 1-6 days after a boxing match (Neselius et al., 2012). Although prior studies did not detect changes in individuals with mTBI, the later study is distinguished from others in that a more sensitive enzyme linked immunosorbant assay was used for quantifying peripheral tau levels of matrix metalloproteinase-9 (MMP-9), an enzyme released from vascular endothelium, neurons, glia, and microglia that can lead to disruption of the blood-brain barrier with associated increases in edema; MMP-9 inhibition reduces blood-brain permeability and neuronal apoptosis (Wu et al., 2020) In addition, the neuronal protein, ubiquitin carboxyl-terminal hydrolase-1 (UCH-L1), is increased in TBI patients with normal CT and serum UCH-L1 and GFAP levels are also associated with poor outcomes (Snyder et al., 2018). It is noteworthy that the findings with UCH-L1 and GFAP received authorization by the U.S. Food and Drug Administration (FDA) of BTI*™* (Brain Trauma Indicator, Banyan Biomarkers). Neurofilament light (NfL) is a neuronal cytoskeletal protein and a marker of axonal damage. In a recent study, plasma NfL levels were increased seven days in a study involving 31 females and 43 males that sustained an mTBI. NfL levels were poorly correlated with early injury ( < 6 hours) (Reyes et al., 2023). Based on these and other findings, it is appropriate to include NfL, tau, and MMP-9, in addition to GFAP and UCH-L1 in a survey of candidate protein biomarkers for TBI severity and outcome.

### 4.2 IL1-β CSF and serum levels and PTE risk

While the cytokine, IL1-β, has been associated with the acute inflammatory response following TBI, its role in late onset PTE had not been studied. Diamond et al. (2014) studied, in a small cohort of late onset PTE patients, associations between IL1-β gene variants (SNPs) and IL1-β protein CSF or serum levels and PTE onset. Late onset PTE patients (one week or greater) were recruited to increase the likelihood that seizures were due to epileptogenic mechanisms and not to other sources. In that study, an increased CSF IL1-β/serum IL1-β ratio was associated with increased risk of PTE relative to heathy controls while multivariate analysis that included the CT genotype for IL1-β gene (*IL1B*) variant rs1143634 indicated that T allele carriers had higher IL1-β serum levels compared to CC homozygotes in the PTE group. The authors suggested that the alterations in IL1-β ratios may represent biological variability resulting from TBI. The directionality of the findings is consistent with current the understanding of proinflammatory mechanisms resulting from TBI but the authors suggested that replication due to small sample size of the PTE and control groups was needed. Additionally, it should be noted that no know function of the *IL1B* variant rs1143634 can account for the findings.

## 5 Consequences of traumatic brain injury

Patients diagnosed with mild to severe TBI develop complications after TBI. It cannot be overemphasized that the financial and psychosocial burdens imposed on patients and family members are enormous. Moderate and severe TBI can incur multiple neurological deficits that may involve the motor, sensory, cognitive, autonomic, gastrointestinal, and genitourinary systems. Impairment of activities of daily living, disability and behavioral changes that can occur after TBI including social isolation associated with anxiety disorders and depression can’t be understated in adults (de Oliveira et al., 2022; Englander et al., 2003; Ponsford et al., 1995; McCrea et al., 2021; Maiden et al., 2020) although children may have a better prognosis (Emami et al., 2017). Pharmacological treatment is directed at the specific neurological deficit. Intrathecal baclofen (Meythaler et al., 1996) and botulinum toxin (Gracies et al., 2015) may be used to reduce TBI-induced spasticity.

Fatigue is another symptom that develops after TBI that is difficult to quantify but is a common complaint. Fatigue is commonly associated with sleep disorders that occur after TBI, but it is difficult to determine the exact nature of this symptom (Sinclair et al., 2014). More research in this area is needed.

Common neurological and psychiatric symptoms that develop after TBI are cognitive i.e. memory impairment, poor attention and/or concentration, and post-traumatic migraine headaches. Affective disorders such as depression and anxiety are also frequently associated with TBI (Hicks et al., 2023; Saravanan et al., 2024). Moderate and severe TBI is also associated with post-traumatic epilepsy. Some other common or less common symptoms that develop after TBI are listed in Table 1. It is also important to acknowledge that these biological changes that arise after injury are typically interactive – e.g., sleep disturbances are often associated with depression and pain. Memory impairment, post-traumatic migraine headaches and post-traumatic epilepsy are further discussed here.

**TABLE 1:** Some of the major medical complications arising from TBI.

Symptom associated with acute TBI	References
Alexithymia	Fynn et al., 2021; Wood and Doughty, 2013
Brain fog	Bell et al., 2023
Coagulopathy	Zhang et al., 2018
Delirium	Maneewong et al., 2017
Dizziness	Wood et al., 2022; Sonstroem et al., 2023
Hearing disturbances and tinnitus	Henry et al., 2019; Clark et al., 2023; Horton et al., 2021
Hypopituitarism	Popovic et al., 2004
Mental fatigue	Johansson and Rönnbäck, 2017
Nystagmus	De Clercq et al., 2017
Pain	Mehalick and Glueck, 2018; Young, 2007
Paroxysmal sympathetic hyperactivity	Compton, 2018; Jang and Kwon, 2022
Pediatric functional neurological symptoms disorder	Landa et al., 2023
Photophobia	Theis, 2022
Psychogenic non-epileptic seizures	Balachandran et al., 2021; LaFrance et al., 2013
Recurrent hypersomnia	Billiard and Podesta, 2013
Sleep disturbances	Lu et al., 2016; Bell et al., 2018; Tosscalino et al., 2021
Suicide	Bryan and Clemans, 2013
Vestibular dysfunction	Skop et al., 2023; Swan et al., 2020
Visual deficits	Armstrong, 2018; Bell et al., 2023; Winkler et al., 2022

### 5.1 Cognitive impairment

Slow informational processing and attentional disorders are frequent complaints across all levels of TBI and include mental slowness and poor performance on complex tasks that correlatewith injury severity (Ponsford and Kinsella, 1992; van Zomeren and Deelman, 1976). Visual and verbal memory tasks are also markedly impaired after severe TBI (i.e., forgetting more frequently and a poor ability to use semantic encoding and/or visual imagery to assist in memory performance). The development of intrusive thoughts is another finding in some TBI patients, although the underlying mechanisms for this condition are unclear (Zec et al., 2001; Geldmacher and Hills, 1997).

Functional magnetic resonance imaging (fMRI) consistently shows increased blood oxygen level dependent (BOLD) activation after moderate to severe TBI in pediatric patients. A subgroup of pediatric patients with slow interhemispheric transfer time after moderate and severe TBI have poor white matter organization, poor cognitive function, ongoing neurodegeneration and poor outcome. Pediatric patients with slow interhemispheric transfer time (those with greater than 1.5 standard deviations of the normal range from healthy controls), showed increased fMRI BOLD activation in the frontal, parietal and occipital regions while performing a working memory task load compared with moderate to severe pediatric TBI patients within 1.5 standard deviations of the normal range of interhemispheric transfer time and healthy controls. This finding suggests that in moderate and severe TBI pediatric subjects with slow interhemispheric transfer time, increased BOLD activation may show more actively engaged neuronal networks that act as a compensatory mechanism during a working memory task load to maintain performance (Olsen et al., 2020).

### 5.2 Post-traumatic headache

Defined as a headache within seven days after a TBI, post-traumatic headaches are common, can be debilitating and challenging to treat. The most common types of post-traumatic headache are migraine-like headache followed closely by tension-like headache (Ashina et al., 2020a). In a recent study, 70% of those with persistent headache after TBI, headaches lasting more than three months, developed migraine-like features within 12 hours after infusion of calcitonin gene-related peptide (CGRP), a neuropeptide that is released from sensory nerves and is involved in pain pathways. Antibodies to CGRP or its receptor are effective treatments for migraine headaches. In an open-label study that targeted the CGRP signaling pathway, 28% of those with persistent post-traumatic headaches had a 50% reduction in the number of headache days per month (Ashina et al., 2020b). Persistent post-traumatic headaches are common after mTBI and observed more in females similar to the gender preference in primary migraine headaches (Seifert and Evans, 2010). Post-traumatic headache symptoms can be migraine-like, tension-like or cluster-like and can occur with sleep disorders, cognitive impairment and emotional disorders (Labastida-Ramírez et al., 2020). Pre-existing headaches prior to trauma, older age (Defrin, 2014), and those with a family history of primary headaches are risk factors for persistent post-traumatic headaches (Ashina et al., 2020a; Sufrinko et al., 2018). Those with a prior history of tension headaches also have an increased frequency of tension-like headaches after trauma (Labastida-Ramírez et al., 2020). It is noteworthy that there are newly discovered microvascular channels in the bone marrow of the skull that may be conduits for inflammatory cells to reach the meninges and to other intracranial structures involved in headache syndromes (Herisson et al., 2018). Imaging showed that these channels may play an important role in migraine (Gao et al., 2021). Despite the similarities in symptoms, gender differences, response to CGRP and effectiveness of migraine therapies that block CGRP signaling, imaging studies consisting of diffusion tensor imaging (DTI), gray matter changes, brain volume, density and thickness show distinct differences between persistent post-traumatic headache and primary migraine headaches suggesting differential mechanisms (Labastida-Ramírez et al., 2020).

### 5.3 Post-traumatic epilepsy (PTE)

Traumatic brain injury is a major cause of acquired epilepsy (Hauser et al., 1991; Guekht et al., 2010). Complex partial seizures or temporal lobe epilepsy consists of between 35 and 62% of seizures from PTE (Zhu et al., 2019; Diaz-Arrastia et al., 2000; Golub and Reddy, 2022). A critical risk factor is injury severity, where moderate and severe TBI are most likely to result in PTE (Annegers et al., 1998; Christensen et al., 2009). In a study from Sweden, PTE risk was evaluated in those individuals ages 18 to 100 after their first hospitalized TBI matched for age and gender. Most of the cohort were male and the preponderance age category was 18–39 years. The most common injury was a mTBI by a fall. About 20% of the individuals in study cohort sustained multiple injury mechanisms and the most severe injury type was focal cerebral injury (4.3%) of males compared with 3.1% of females. The median time of the development of epilepsy was one year and the median age was 61 years. Their results showed that the 10-year risk for PTE was 12.9% for focal cerebral injuries, 8.1% for diffuse cerebral injuries, 7.3% for extracerebral injuries, and about 2% for skull fractures and mTBI while the 10-year epilepsy risk for controls was 0.9%. The severity of injury increased the risk of PTE where focal cerebral injuries incurred the greatest risk of epilepsy followed by diffuse cerebral injuries. Other risk factors included male gender, age, those on mechanical ventilation, and having a seizure during the hospitalization. The overall 10-year seizure risk for any TBI was 4% and it was 0.9% in controls (Karlander et al., 2021). Individuals with skull fracture, severe and mTBI had a higher risk of PTE compared with age and sex-matched control groups whereas men and those that had mixed types of cerebral hemorrhages carried the highest risk of PTE; the greatest risk was in the first year. Associated factors included younger age, low income, living in more rural areas, mental disorders, liver cirrhosis, migraine headaches, and dialysis status (Yeh et al., 2013). In a retrospective study in Sweden, the relative risk of PTE was determined in 1,885 cases after TBI over an eight-year period compared with over 15,000 controls. The relative risk was 2.0 in mTBI cases. The relative risk was almost 3-fold and 2-fold higher in cases with a brain contusion or intracerebral hemorrhage. In those cases with a contusion and intracerebral hemorrhage, the relative risk was almost 7-fold higher compared with mTBI without a lesion on structural brain imaging. The risk for PTE was highest during the first six months after a mild or severe TBI but remained elevated for over 10 years for any TBI severity (Mahler et al., 2015). In another study, approximately one-third of those with severe TBI developed PTE. This PTE group had a two-fold increase in the vegetative and severely disabled category by the Glasgow Outcome Scale at two years indicating a worse outcome over time (Pease et al., 2023). Analysis of more than 57,000 TBI subjects registered in Denmark hospitals showed that TBI was associated with low-income status and poor general health. PTE increased with the magnitude of injury and was especially high for those in the age group category of 40–59 years. The risk of PTE occurred two-four years after TBI (Lolk et al., 2023). The results of the Danish study correlated well with another study that performed a systematic review and meta-analysis (Sui et al., 2023).

Evidence is accumulating that predictors and biomarkers of PTE may be used in the near future. A multicenter, retrospective case-controlled study (64 cases, 64 controls) of adults 18 years or older, who suffered either a closed head or penetrating TBI without a history of seizures or epilepsy and did not develop PTE were compared with subjects that developed PTE. All patients were monitored about two weeks and twelve months after injury by electroencephalography (EEG). Patient groups were matched based upon sex, GCS and age. PTE was defined as an unprovoked seizure that occurred one to twelve months after TBI. Subjects who had a penetrating TBI, a subdural hematoma, or a skull fracture were all at increased risk for developing PTE. Individuals with a greater burden of early electroencephalographic seizures, epileptiform discharges, lateralized or generalized periodic discharges, lateralized rhythmic delta activity and asymmetrical delta frequency were all associated with an increased risk of PTE. Interestingly, generalized rhythmic delta activity without epileptiform activity showed a negative association with PTE (Chen et al., 2023).

### 5.4 TBI and risk of neurodegenerative disorders

#### 5.4.1 Dementia

A history of TBI can increase one’s risk of all-cause dementia and subtypes such as Alzheimer’s Disease at any severity level. Many large studies around the globe have shown an increased risk of dementia after TBI (Li et al., 2017; Fann et al., 2018; Perry et al., 2016; Nordström and Nordström, 2018; Raj et al., 2022; Gardner et al., 2023). TBI is associated with an increased risk for Alzheimer’s Disease (Mortimer et al., 1991; Fleminger et al., 2003), Parkinsonism (Jafari et al., 2013; Balabandian et al., 2023) and CTE (Stein and Crary, 2020; Kelly et al., 2023; Omalu, 2014; Bieniek et al., 2021). Recently, a study from the Veterans Health Administration (VHA) showed an elevated risk for dementia in mTBI as well as in moderate-severe TBI individuals. This was a retrospective study that began in 2001 and ended in 2014; the total number of subjects was 178,779 in those with or without a history of one or more TBIs. TBI was diagnosed using the Department of Defense or Defense and Veterans Brain Injury Center criteria. mTBI was divided into mTBI with and without loss of consciousness (LOC). The average age in both TBI groups was 49 years, nine percent of the individuals were women, 73% were Caucasian, 16% were African American and 1.6% were Hispanic. Fifty-two percent were college educated and all levels of income were represented in the study. The two sources used to obtain TBI clinical information came from the Comprehensive Traumatic Brain Injury Evaluation (CTBIE) database and the National Patient Care Databases (NPCD) which come from VHA inpatient and outpatient medical appointments. The investigators used ICD-9 codes to define dementia. Regarding associated medical conditions, 11% had diabetes mellitus, 11% had hypertension, 19.4% had a mood disorder, and 11% had post-traumatic stress disorder (PTSD). Follow-up was approximately 4 years. The results of their study showed an adjusted increase in risk of 2.4 in developing dementia for mTBI without LOC, 2.51 for mTBI with LOC, 3.19 for mTBI with LOC status unknown and 3.77 for moderate to severe TBI (Barnes et al., 2018). The results of this study are similar to two other studies showing a small but significant risk of developing dementia particularly those that sustain an mTBI over the age of 55 years (Gardner et al., 2014). A major concern with many of these studies is that they are based on retrospective ascertainment of mTBI. Large well-controlled prospective studies are greatly needed to determine the effect of mTBI on the development of dementia. Genetic and other factors are predicted to modify risk of developing dementia. As stated above, *APOE4* is linked to poorer memory performance and outcome after TBI and is a risk factor for late-onset AD (Frieden and Garai, 2013).

#### 5.4.2 Parkinson’s disease (PD)

Traumatic brain injury with LOC is associated with a significant risk of Parkinson’s Disease (PD). In those individuals with a TBI and less than or more than one hour LOC, the adjusted hazard risk with a 95% confidence interval was 0.66 and 3.56, respectively in the Adult Changes in Thought (ACT) study. Progression of PD signs had an adjusted odds ratio of 1.75 that was based upon eight scoring ranges derived from 26 items of the Unified Parkinson’s Disease rating Scale. TBI with LOC less than one hour was associated with an increased risk of Lewy bodies in the frontal or temporal cortex. Curiously, this large dataset consisting of the Religious Orders Study (ROS), ACT, and the Memory and Aging Project (MAP) did not find a relationship between TBI with LOC up to one hour or after one hour and mild cognitive impairment (MCI), Alzheimer’s Disease (AD) and dementia. There was no relationship between TBI with LOC and MCI in the ROS and MAP. Additionally, *APOE* genotype did not alter the lack of relationship between dementia and TBI and there were no statistically significant increases in neurofibrillary tangles or neuritic plaques in the brains in this cohort (Crane et al., 2016). Unfortunately, further characterization of the TBI subjects and the presence of Lewi bodies in substantia nigra neurons was not performed and the percent loss of substantia nigra neurons in the midbrain was not calculated to confirm the diagnosis of PD. To investigate the relationship between TBI and PD, a meta-analysis of 22 peer-review publications including 19 case-controlled studies, two nested case-control studies, and one case control study was performed by investigators in Canada. This group used the Newcastle-Ottawa Scale (NOS) to determine the quality of studies based upon the exposure or outcome, the selection of subjects, and comparability of the groups of subjects (Jafari et al., 2013). Studies included subjects with a confirmed diagnosis of PD using established criteria by trained staff and/or neurologists and/or from a brain bank and history of TBI where a clear definition of TBI was stated in the article using the presence or absence of LOC. However, TBI injury severity and the number of TBIs were not stated in most of the articles used for the meta-analysis. The time of the TBI prior to the onset of PD was from an unknown period of time to more than 30 years prior to PD diagnosis. Results from the meta-analysis using only those studies that reported adjusted or matched odds ratio showed a significant association in those individuals that had a TBI with an LOC and PD (Jafari et al., 2013). The possible association between mTBI and PD remains uncertain with studies showing disparate findings (Rugbjerg et al., 2008; Seidler et al., 1996; Kuopio et al., 1999; Bower et al., 2003; Goldman et al., 2006). A recent retrospective study reported a 56% increase risk of PD after mTBI with LOC in veterans and an 83% PD risk in veterans diagnosed with moderate to severe TBI with LOC, alteration of consciousness or post-traumatic amnesia (Gardner et al., 2018). In summary, men were twice as likely to be diagnosed with PD and men account for the vast majority of TBIs but more prospective studies are needed to confirm the relationship between head trauma and PD.

#### 5.4.3 Chronic traumatic encephalopathy (CTE)

Punch-drunk syndrome first described by Martland (1928) (see Parker, 1934), was the original description of individuals that developed signs and symptoms of a progressive neurodegenerative disorder due to repetitive mTBIs from boxing. Since these initial descriptions in boxers, non-boxer atheletes and military veterans that were exposed to repetitive blast or a single blast mTBI (Omalu et al., 2010) demonstrated the same pathology in the brain called CTE (Geddes et al., 1999; Omalu et al., 2005; Omalu et al., 2011; Saing et al., 2012; Goldstein et al., 2012; Smith et al., 2013, 2019; Stewart et al., 2016; Lee et al., 2019; Buckland et al., 2019). In one case, the patient was a 44-year-old retired National Football League (NFL) player who started playing football in high school and continued through college and spent nine years in the NFL. He had a reputation for being a hard hitter and aggressive player. He did not abuse steroids or other illicit drugs but admitted to family members that he lost count of his concussions after age 15. Following retirement, he made bad investments noting that his judgement was poor and began having memory difficulties. His working memory was also impaired as he would lose his train of thought while speaking during a conversation; he would forget about plans, appointments, and schedules. He got lost driving home once and had to call a good friend to help him find his way home. He became socially isolated and would get easily angry if things didn’t go his way and would have exaggerated emotional responses to minor events. He had insomnia and at times would work without sleep. He was depressed and had suicidal ideations and then became paranoid, committing suicide 11 years after retiring from the NFL. Regarding his *APOE* genotype status, he had two copies of *APOE3*, so it is unlikely that genetic variation at *APOE* contributed to the development of CTE in this individual. However, CTE risk is increased in older men (greater that age 65) who participated in contact sport participants and or were military veterans (Atherton et al., 2022).

Like other neurodegenerative disorders such as AD and PD, CTE can only be definitively diagnosed at autopsy. Autopsy findings in cases of CTE show a primary tauopathy that can be together with amyloidopathy and TDP proteinopathy, TDP-43 is an RNA binding protein essential in development and function (Sephton et al., 2012). Neuritic threads (NTs) and neurofibrillary tangles (NFTs) are the hallmark findings of the primary tauopathy at autopsy (Omalu, 2014). The CTE tauopathy consists of phosphorylated tau fibrils in neurons and astrocytes as well as their processes which is distinct from the NFTs of AD. Clusters of perivascular neuronal and astrocytic phosphorylated tau are considered pathognomonic for CTE. Sparse distribution of NFTs in the frontal, temporal, parietal, insular and septal cortices are frequently found in the brains of CTE (McKee et al., 2016). Currently, the evidence suggests a causal relationship between repetitive head injury and CTE (McKee et al., 2023).

## 6 Medical management of TBI

Traumatic brain injury can occur under a variety of conditions including contact sports, transportation-related (bicycle riding, motor vehicles etc.), falls, penetrating injuries i.e. bullet, shrapnel or by blast. Any direct impact to the head or blow/jolt to the head associated with rapid acceleration and deacceleration can result in a TBI. Repetitive mTBI, subconcussive (no brain injury symptoms) or concussive brain injury, occurs usually as a result of contact sports or military service; these individuals may develop CTE and have a worse prognosis (Lindquist et al., 2017; Bailes et al., 2013; Fehily and Fitzgerald, 2017). Neurological deficits and level of disability are major determinants of prognosis. There are many behavioral measures that can be employed to determine various clinical expressions of TBI including the Rancho Los Amigos level of cognitive function (Cifu et al., 1997), a disability rating (Rappaport et al., 1982), agitation behavior scale (Bogner et al., 1999) as well as many others. Typically, a complete neuropsychological battery is performed on symptomatic TBI patients referable to cognition. Behavioral symptoms can be evaluated and treated through counseling, cognitive behavioral therapy, or psychiatric services.

Primary injury results in direct damage to the skull and/or underlying brain tissue. Secondary injury involves the cascade of molecular and cellular mechanisms involved in either degeneration of the skull and/or brain tissue. The alterations that can occur after the primary injury involve the initial stretching of axons and cell bodies, white matter tract disruptions, indiscriminate permeability of sodium, calcium and potassium ions across a damaged plasma membrane and dysfunctional ion channels, overactivation of ionotropic excitatory amino acid receptors leading to glutamate-mediated excitotoxicity, blood-brain-barrier breakdown, oxidative stress, lipid peroxidation, inflammation, mitochondrial failure, ischemia, apoptosis and necrosis (Werner and Engelhard, 2007; McGinn and Povlishock, 2016; Kaur and Sharma, 2018).

### 6.1 Diffuse axonal injury (DAI)

Up to 50% of all TBIs involve DAI. DAI can manifest from cognitive impairment to coma (Smith et al., 2003; Johnson et al., 2013). DAI can occur in mild to severe TBI. Pathological analysis showed axonal cytoskeletal breaks from mechanical shearing force, disruption of axonal transport of essential nutrients, swelling and breakdown of axons (Adams et al., 1989; Povlishock, 1992; Povlishock and Becker, 1985; Povlishock and Katz, 2005). Continued axonal neurodegeneration may occur after acute TBI and DAI but has been found years later (Chen et al., 2009) and can lead to disconnection syndromes (Monaco et al., 2013; Johnson et al., 2013; Sharp et al., 2014). The LOC from the initial brain injury is likely due to DAI where examination of the brain would reveal widely distributed axonal injury in the cortex, cerebellum, and brainstem. Small cerebral lesions at the gray-white junction and midline structures are vulnerable to shear forces (Adams et al., 1989). That is, swollen axons could be recognized across all magnitudes of brain injury (Oppenheimer, 1968). Rapid acceleration-deacceleration and impact brain injuries all showed DAI (Gennarelli et al., 1982).

The major innovation today is that DAI in human brain can be identified by magnetic resonance imaging (MRI) in the diffuse tensor imaging (DTI) mode (Zimmerman et al., 2021). DAI is graded according to Adams et al. (1989) where grade I is axonal injury in the cerebral hemispheres and in particular at the gray-white junction, grade II is axonal injury in the corpus callosum, and grade III is axonal injury in the brainstem. A recent study with a five-year follow-up sought to determine the clinical correlation of DAI and outcome. Subjects with a documented acute brain injury on head computer tomography (CT) were eligible for the study. This is the largest long-term study undertaken to investigate the relationship between DAI and outcome. A total of 311 patients were enrolled in the study excluding patients with penetrating head injuries, epilepsy, a psychiatric disorder, and those who did not speak English. A brain MRI was obtained within two weeks of injury. Results showed that 56% of the patients had DAI where 25% had Grade I, 18% had Grade II, and 13% had Grade III. Most of the patients were white males and the ages ranged from 23 to 57 years. The Emergency Department GCS range was 3–14 and the Marshall Head CT score was 2 (midline shift up from 0 to 5 mm, cisterns are not compressed, and absence of high or mixed density lesion) (Maas et al., 2005) Private insurance was significantly correlated with a higher Glasgow Outcome Score extended (GOS-e). Hospital length of stay ranged from 5 to 21 days. Those patients with DAI had a worse functional status on discharge. However, there was no association between DAI and long-term functional outcome or quality of life. No association was found between the presence or absence of DAI on MRI and the 3-year mortality. Age, injury severity score, number of days on the ventilator, and inability to follow commands were all significantly correlated with the three-year mortality. These investigators also reported that despite a worse prognosis in those that had DAI in the corpus callosum, one-third of patients had a good recovery (Humble et al., 2018). It has been reported that the DAI lesion and location after moderate-severe TBI could be used as a prognostic indicator to predict functional outcome (Moen et al., 2014). Taken together, early MRI imaging to detect DAI after moderate-severe TBI may not have prognostic value on long-term functional outcome or quality of life and awaits further research.

### 6.2 Pre-hospital management of TBI

The goal of the pre-hospital TBI evaluation is to assess patients for life-threatening injuries, limit secondary brain injury and to rapidly transport subjects to the hospital. In the field and at the hospital, it is important to remember the ABC’s in medicine: airway, breathing and circulation. During transportation to a hospital, assessment of the patient for signs of herniation must be performed including asymmetry of pupillary size, reaction to light and motor signs including decerebrate posturing as well as blood pressure, pulse, and respiratory rate. Mental status exams must be performed using the Glasgow Coma Scale (GCS) as a guide because a reduction in the GCS of two or more points is associated with a poor outcome (Servadei et al., 1998). Rapid assessment of external or internal hemorrhage, spine and other fractures, brain intraparenchymal hemorrhage, contusion, epidural, subdural and subarachnoid hemorrhage, and a neurological exam are performed in concert with a head CT and appropriate laboratory analysis including blood sugar. The GCS is a good tool to use for overall neurologic assessment and consists of best eye opening, motor and verbal responses. The GCS is used to classify TBI and for triage of patients. A GCS of 13–15 defines mTBI, a GCS of 8–12 defines moderate TBI and severe TBI is defined as a GCS score of 3–8 (Zafonte et al., 1996).

### 6.3 In-hospital management of TBI

Medical management of TBI in the hospital depends on the magnitude of brain injury, existence of trauma to other parts of the body, types of injury and life-threatening complications. Complications such as a rise in intracranial pressure secondary to a structural abnormality such as a hematoma o; r intraparenchymal hemorrhage, or diffuse cerebral edema require emergent treatment. Patients who have clinical signs of increased intracranial pressure such as papilledema or acute onset pupil asymmetry after TBI may require intracranial pressure (ICP) monitoring in an intensive care unit setting. Cerebral edema is a major predictor of adverse outcome and decreased cerebral perfusion pressure from an increased intracranial pressure or cerebral hypertension resulting in secondary cerebral ischemic damage and in-hospital mortality (Jha et al., 2019; Hudak et al., 2014; Iaccarino et al., 2014; Beaumont and Marmarou, 1999; Tucker et al., 2017). Standard-of-care treatment of cerebral edema include hyperventilation, hyperosmolar agents like hypertonic saline and mannitol or decompressive craniectomy to reduce intracranial pressure but it is uncertain how these treatments affect cerebral edema as it relates to intracranial pressure and outcome (Gottlieb and Bailitz, 2016; Jha et al., 2019).

### 6.4 Medications and other treatments

#### 6.4.1 Current and under investigation

The first report on concussive injury in the experimental literature (Brown, 1799) is a case report of the treatment two hours post injury and subsequent death of a man who fell from his horse. The following medications were given as a mix: potassium tartrate, lavender, various purgatives, and other herbals. There are currently no medicines specifically designed or approved for use in TBI victims or for prophylactic treatment. There are instead, two major uses of existing drugs after TBI: these are medicines that aim to reduce additional biological impairment and those used to treat specific symptoms or symptom classes. In the first case, antiseizure drugs, coma-inducing medicines and diuretics are often used post injury. For symptom management, a host of drugs are prescribed for headache, pain, blood clotting, seizures, fatigue, and mood and anxiety. Of the new trends in drug discovery for TBI, a 2019 report found that of 203 intervention clinical trials, 34% were for acute physiological disruption or intracranial pressure, 23.6% studied neurotransmitter systems, and 17.2% studied behavioral interventions and stimulation methods. Methods to influence metabolic functions constituted 9.9% of the clinical studies, and inflammatory and immune function were the targets of 7.4% of the trials (McGuire et al., 2019).

As of March 6, 2024, there were 1736 total trials listed in ClinicalTrials.gov for TBI including completed trials and those currently recruiting. A partial listing of trials in which compounds were part of the study protocol is shown in Table 2. The table illustrates categories of post-TBI sequalae that are being targeted for the discovery of new treatments such as cognitive impairment, headache, and PTE. The diversity of pharmacological drug classes being interrogated illustrates the complexity of the disorder and its secondary consequences that require medical attention and the current gaps in our understanding of mechanisms. Diversity of mechanisms of action under investigation also point to the fact that TBI patients require medical help, and we are still looking for better ways to provide this. Clinical investigation allows hypotheses to be tested at the patient level. In Table 2, it can be seen that several potential ideas are being studied: neuroprotection (NMDA receptor antagonists and others), neurogenesis (as with NGF and GF), and tau and beta-amyloid hypotheses.

**TABLE 2:** Compounds listed as being evaluated in clinical trials for TBI^1^.

Compound	Pharmacological class^2^	Clinical study
Valproate (6)	GABA PAM	PTE−Prophylaxis, Phase 3 Neuroprotection Mood and alcohol use
Progesterone (5)	GABA PAM	Neuroprotection Neurological symptoms Recovery
Allopregnanolone (3)	GABA PAM	PK and Glasgow score
Midazolam (7)	GABA PAM	Cerebral metabolism Intracranial inflammatory responses Oxidative stress Sedation
Pregnenolone (3)	GABA NAM	Cognition, anxiety, depression PTSD and pain
Topiramate (3)	GABA PAM	PTE
Levetiracetam (8)	Anticonvulsant	PTE
Carbamazepine (1)	Anticonvulsant	PTE Irritability and aggression
Biperiden (2)	M1 antagonist	PTE
Sumatriptan (2)	5-HT agonist	Headache
Amitriptyline (2)	Antidepressant	Headache
Atomoxetine (1)	NE uptake inhibitor	Cognition/Attention
Methylphenidate (10)	DA uptake inhibitor	Cognition and neurocognition Attention
Sertraline (4)	5-HT uptake inhibitor	Depression
Citalopram (4)	5-HT uptake inhibitor	Depression Anxiety
Buspirone (4)	5-HT agonist	Irritability, depression
Propranolol (10)	NE antagonist	Cardiovascular Sympathetic hyperactivity
d-Cycloserine (2)	NMDA antagonist	Cognition
Amantadine (13)	NMDA antagonist	Irritability and aggression Recovery Neurological outcomes
Memantine (5)	NMDA antagonist	Verbal learning and memory
Ketamine (16)	NMDA antagonist	Intracranial pressure and brain tissue oxygenation and neuroprotection PTSD and MDD
Dexmedetomidine (10)	Novel sedative	Intracranial pressure in severe TBI
Rivastigmine (3)	Cholinesterase inhibitor	Cognitive function
Cannabidiol (7)	Cannabinoid	Neuropsychiatric symptoms, PTSD, and cognition
Ronopterin (2) (VAS203)	NO synthase inhibitor	Inflammation and oedema PK and intracranial pressure
Nitric oxide (3)	Oxygen enhancement and other	Cerebral metabolism
Erythropoietin (8)	Red blood cell hormonal stimulator	Survival and quality of life
ABX-101 (1)	Thyroid hormone receptor beta agonist	Glasgow Outcome Scale-Extended
NGF (7)	Neurotrophic factor	Neurological functions Anxiety and depression
Growth factor and related (5)	Neurotrophic factor	Brain function
SLV334 (1)	Endopeptidase inhibitor−neuroprotectant	Safety Terminated study
Botulinum toxin type A (18)	Nerve toxin	Spasticity and pain
DHA (7)	Omega-3 fatty acid	Brain health biomarkers
Minocycline (2)	Antibiotic	Glasgow outcome scale-extended Disability rating scale
NeuroAid II (2) (MLC901)	Natural product derived	CNS vital signs and executive function
Simvastatin (2)	Statin-type cholesterol lowering agent	ICU duration and Glasgow coma scale Tau and A-beta CSF levels
PNT001 (1)	Monoclonal antibody to the cis isomer of tau phosphorylated at threonine 231	Ph1 study – terminated for administrative reasons

^1^The compounds compiled here were all generally listed in more than one clinical trial (number of trials are in parentheses). Data compiled from a March 6,2024 search of ClinicalTrials.gov.

^2^Many compounds have multiple mechanisms of action. 5-HT, 5-hydroxytryptamine or serotonin; DA, dopamine; DHA, docosahexaenoic acid; NAM, negative allosteric modulator; NE, norepinephrine; PAM, positive allosteric modulator; PK, pharmacokinetics; PTE, post-traumatic epilepsy.

A number of these trials were for biomarker analysis, repetitive transmagnetic stimulation (rTMS), and other non-drug interventions (Table 3). Although biomarker studies are not shown in this table, they are valuable for diagnostics and guiding research into improved therapeutics. The data in Table 3 emphasize several important aspects of clinical research in TBI. First, there are multiple biological systems that require assistance (balance, walking, planning, fatigue, neuropsychiatric symptoms). Second, in addition to the medication therapies under clinical investigation (Table 2), Table 3 shows a multitude of approaches to solving symptom problems post TBI. The diversity of interventions being studied suggests that the symptoms require different interventions and the ability of a clinical trial to test biological hypotheses (e.g., does a ketogenic diet help with neurocognition?).

**TABLE 3:** Non-compound related interventions listed as being evaluated in clinical trials for TBI^1^.

Intervention	Clinical study endpoints
Aerobic exercise and related (206)	Post concussion symptoms questionnaire
Light therapy (67)	Sleep, cognition, and fatigue
Transcranial direct current (40)	Word association
Virtual reality treatment (38)	Balance, pain
rTMS (35)	Cognitive measures and quality of life, depression, and headache
Executive function training (32)	Virtual multiple errands test
Hyperbaric oxygen (22)	Disability rating scale and Glasgow outcome scale-extend
Acupuncture and variants (17)	Spasticity, mobility, balance
Hypothermia (11)	Glasgow outcome scale
Yoga (10)	Balance, balance confidence, body responsiveness, pain, physical ability, and quality of life
Deep brain stimulation (8)	Improvement of brain function
Autologous bone marrow mononuclear cells (6)	Neurological events and global functioning
Ketogenic diet (4)	Neurocognitive outcome
CN-NINM (3)	Sensory organization test and Neurobehavioral symptom inventory
Neurofeedback-enhanced mindfulness meditation (1)	Neurobehavioral symptom inventory
Locomotor training (1)	Gait speed and endurance

^1^The interventions compiled here were all generally listed in more than one clinical trial (number of trials are in parentheses). Data compiled from a March 6, 2024 search of ClinicalTrials.gov. CN-NINM, cranial-nerve non-invasive neuromodulation; rTMS, Repetitive transmagnetic stimulation.

#### 6.4.2 New vistas

##### 6.4.2.1 GABA

The prominent role of β-aminobutyric acid (GABA) neurotransmission in the control of inhibitory processing in the CNS makes it not surprising that GABA receptors (GABARs) are also a primary focus on therapeutics in TBI (Table 2). A recent review of the literature concluded that GABA and its receptors are critical to the pathophysiology deriving from TBI. Unfortunately, it was suggested that more directed clinical research efforts are required to help deliver improved GABA medicines to TBI patients (Witkin et al., 2024b). In particular, it has been recommended that investigators think about the potential of novel GABAR potentiators for therapeutics. In particular, GABA_*A*_R potentiators with reduced sedative liability (see Witkin et al., 2022; Witkin et al., 2024b for details and discussion).

##### 6.4.2.2 AMPA receptor antagonists

α-Amino-3-hydroxy-5-methyl-4-isoxazolepropionic acid (AMPA) is the principal neurotransmitter for driving fast excitatory signaling in the CNS. Although its role in physiological and pathophysiological conditions of the CNS is unequivocal, the drugs that modify AMPA receptors (AMPAR) function are typically not tolerable. This section will discuss the potential of AMPAR drugs for TBI therapeutics.

Pain is one area of immense therapeutic need after TBI. AMPAR antagonists have been shown to attenuate chronic pain after nerve injury as such might also be viable treatments for TBI-induced pain states. Perampanel is the only AMPAR antagonist approved for use in humans (epilepsy) and has been proposed as an intervention in other neurological disorders such as neurodegenerative diseases (Perversi et al., 2023). Its efficacy in preclinical pain models is well documented (Khangura et al., 2017; Hara et al., 2020; Witkin et al., 2023) including chronic pain induced by spinal cord injury (Witkin et al., 2024a) and importantly perampanel has also been reported to dampen chronic pain in humans (Chang and Park, 2021). Therefore, we suggest that clinical scrutiny of perampanel in TBI patients. As noted in Table 2, no study on this drug or mechanism is under current clinical evaluation.

Other possibilities should arise for this mechanism in the near future. LY3130481, now ES-481, is being developed for pharmacoresistant epilepsy by ES Therapeutics Australia Pty Ltd. This compound was discovered to overcome the sedative and ataxic liabilities of perampanel (Youn et al., 2018). Although it is an AMPAR antagonist, LY3130481 blocks only a subset of AMPARs (those associated with the auxiliary protein TARP-g-8 and thereby bypassing AMPARs in the cerebellum that are responsible for ataxia (Gardinier et al., 2016; Kato et al., 2016; Kato and Witkin, 2018). LY3130481 displays a much-improved side-effect profile compared to perampanel while continuing to engender high efficacy against seizures (Kato et al., 2016) and pain (Knopp et al., 2019) in rodent models.

To date, perampanel, the only medically approved AMPA receptor antagonist, is not generally prescribed for TBI cases. However, it is used as a antiseizure medication as per its approved use. Its use in pain states post TBI will require focused clinical scrutiny. Further, the role of AMPA receptor antagonists as neuroprotectant agents (da Silva et al., 2024; Sharma et al., 2023; Yang et al., 2021) enables another area of future clinical evaluation for use in TBI patients.

##### 6.4.2.3 AMPA receptor potentiators

In addition to being blocked by antagonists, AMPARs can also be augmented in function by potentiators. Amplification of excitatory CNS function could be of value for TBI cases in at least two major ways. First, as the CNS remodels after injury as discussed previously, the facilitation and/or direction of remodeling could be useful for repair of function. Secondly, AMPAR potentiation induces neurogenesis factors that could help to regain lost neural net connectivity.

The basic mechanism of neural remodeling or neural plasticity (see Makin and Krakauer, 2023 for arguments against this) involves the process of long-term potentiation (LTP) whereby one neural trace can increase the synaptic strength of a subsequent trace (Malenka and Nicoll, 1999). AMPAR potentiators facilitate the formation of LTP by Strong synaptic activation of AMPARs and resulting depolarization removes the magnesium block of N-methyl-D-aspartate (NMDA) glutamate receptors. Calcium influx through open NMDA receptors, further boosted by the activation of calcium channels (Cifuentes et al., 2004), triggers a host of intracellular messaging systems which lead to the phosphorylation of AMPA receptors and an increase in their surface expression (Huganir and Nicoll, 2013). These functional changes are followed by morphological changes such as synaptic sprouting and synaptogenesis (Lynch, 2004; Sumi and Harada, 2020).

AMPAR potentiators also known as ampakines trigger LTP through their initial activation of AMPARs as such have been shown to improve learning and memory and other synaptic plasticity events in the CNS (Lynch, 2004; Arai and Kessler, 2007). Recent preclinical studies have documented the therapeutic value of AMPAR potentiators in some of the secondary symptoms arising from spinal cord injury (Witkin et al., 2024a).

AMPAR potentiators also upregulate BDNF that then activates the TrKB receptor (see Section IV and Table 2 for additional reference). Although a number of methods have been studied to promote the induction of BDNF, they are incumbered by delivery issues and other complexities (Martínez-Gálvez et al., 2016; Mantilla et al., 2013; Marchionne et al., 2020, Sieck et al., 2021; Crowley et al., 2019; Ji et al., 2015), ampakines are small molecules that provide the drug-like method of enhancement. Ampakines enhance BDNF, facilitate LTP, and create a milieu for synaptic plasticity changes to arise (Simmons et al., 2009; Simmons et al., 2011; Lauterborn et al., 2016). For example, motor recovery after stroke has been positively impacted by ampakines (Clarkson et al., 2011) and have shown promising results in preclinical studies of spinal cord injury (Witkin et al., 2024a).

As with AMPA receptor antagonists, AMPAR potentiators are also not in the routine prescription package for TBI. However, the pharmacological properties of these potentiators to regulate neuroplasticity changes and improve impaired cognitive function (discussed above) provide important areas of impact in TBI where these compounds could be explored in the clinic. The recent studies showing improvements in function after spinal cord injury with AMPAR potentiators (Witkin et al., 2024a) will also likely invigorate further research in these domains for TBI patients. Fortunately, there are such compounds that are approved for clinical study such as CX1739 (see Witkin et al., 2024a) that can be used for research in patients.

##### 6.4.2.4 Apolipoprotein E (ApoE)

Although their induction has a unique distal trigger, neurodegenerative diseases such as amyotrophic lateral sclerosis (ALS), Parkinson’s Disease, and Alzheimer’s Disease ultimately result in neurodegeneration that is part of the biological process post TBI. Thus, it is likely that we can gain great insight into at least some aspects of TBI by our current and ongoing building of the knowledge base of these common neurodegenerative mechanisms. Indeed PNT001, a monoclonal antibody to the cis isomer of tau phosphorylated at threonine 231 is listed as a treatment under clinical investigation for TBI patients (Table 2). Clinicians and patients await the outcome of such studies to guide potential future treatment options.

ApoE, one of the major proteins associated with Alzheimer’s Disease (Serrano-Pozo et al., 2021) and represents one example of where crosstalk with a neurodegenerative disease might provide a novel approach to TBI therapeutics (Laskowitz and Van Wyck, 2023). TBI generally involves DAI (Section VI.A). Damaged axons have limited ability to regenerate markedly hindering functional recovery post TBI (Tedeschi et al., 2017). ApoE might be a target for therapeutic intervention to help facilitate axonal regeneration and ultimately synapse formation. Changes in ApoE have been linked to TBI in patients (Mayeux et al., 1995; Sorbi et al., 1995; Nkiliza et al., 2023). ApoE is a plasma protein associated with lipid and cholesterol transport and linked to neuronal injury (Mahley, 2016; Mahley and Huang, 2012). In addition to its involvement in key biological processes associated with TBI such as neurogenesis and inflammation (Hong et al., 2015; Zhong et al., 2017), ApoE has been shown to directly linked to the processing of neurite outgrowth (Li et al., 2010; Hashikawa-Hobara et al., 2011).

Huang et al. (2020) provided data affirming the potential of ApoE as a target for axonal regeneration in TBI. They used a controlled-cortical impact (CCI) model of TBI in mice. Comparing ApoE -/- to ApoE +/+ mice, they observed lower degrees of axonal regeneration and biochemical markers thereof in the ApoE deficient mice; other proteins were not altered (e.g., myelin basic protein). The reduction in axonal regeneration was associated with deficits in functional recovery at the whole animal level. Additional confirmation of the ApoE hypothesis derived from the result of studies in primary neuronal cultures where treatment of injured neurons with recombinant ApoE protein facilitated axonal outgrowth and growth cone formation in injured neurons. Huang et al. (2020) further identified Dab1 activation as a key molecular process by which ApoE generates its beneficial effects on both biochemical markers of axonal health and functional recovery. Although this area of research is not mature, the encouraging findings showing a role of ApoE in the biological sequalae seen in TBI provides impetus for further research to help discover ApoE modulators that positively impact TBI prognosis.

##### 6.4.2.5 Other innovations

Some of the novel drug interventions that are being explored for the therapy of TBI have been summarized in Table 2 (some novel drugs such as MLC901 or SLV334). Non-drug innovations being studied currently are listed in Table 3. These include special diets and supplements, acupuncture of various types, specific exercise regimens, and oxygen and light therapies. A host of electric stimulation methods including deep brain stimulation are also highlighted in ongoing clinical trials (Table 3).

A number of specialized approaches to symptom improvement by neural monitoring (Mladinic and Nistri, 2013) and stimulation (Ho et al., 2014; Lorach et al., 2023). Computer interfacing of CNS neurons is also an emerging area of dedicated inquiry (Levett et al., 2023). Methods of cell transplantation to promote neural regeneration (e.g., neural stem cell transplantation) are being studied fairly rigorously (Kadoya et al., 2016; Poplawski et al., 2020; Zheng and Tuszynski, 2023) and some findings in this area have enabled clinical study (Zipser et al., 2022).

## 7 Discussion

As the review of the literature makes clear, TBI is prevalent, pervasive, and extremely harmful to patients, their families, and society. We know much about the disorder having researched this for over a century. However, as with many diseases, we know many facts about TBI without a heuristic understanding or appreciation for best practices for patients that have not improved markedly from one hundred years ago of either preventing or moderating symptoms. This is not a criticism, but the natural progress of this science.

Increased medical research on TBI will lead to improved treatment options both prophylactic and symptomatic. The work in the area of Alzheimer’s disease is arguably a good case for comparison. Research into this disorder had been ongoing for about a century as has that of TBI and yet the amount of research devoted to Alzheimer’s disease is 309% greater than that of TBI research. It was not until 1984 that disclosures on beta amyloid started to populate the literature in relation to Alzheimer’s disease. This area of scientific inquiry was grasped firmly, and although skepticism of underlying pathophysiology confronted investigators, huge investments ensued, and progress was made. As a result of this intellectual and financial investment, recent drug approvals for the treatment of AD progression have been granted. The newest drug on the market for the treatment of AD, donanemab, slows clinical progression in those patients with early (mild) AD (Sims et al., 2023).

A similar situation exists for TBI research, with a recognition that the clinical heterogeneity and complex pathophysiology of TBI is the result of it being a condition, and not a disease. This complicates differentiating the pathological, clinical, pysiological, genomic, and proteomic responses of the brain following injury. Additionally, our diagnostic tools are relatively crude, which challeges how to determine treatment options, treatment response, and ultimately patient health outcomes. To date, there has been an inability to predict outcomes for TBI patients. To better stratify patients will require the collection of high-quality data in large cohorts of both TBI patients, patients with non-TBI injury, and healthy controls. It is critical that improved diagnosis be the first step toward identifying patient groups that may demonstrate the benefit of new therapies.

Identification of specific subsets of patients will ensure that these patients are matched to appropriate therapies. This must be based on improved characterization of brain injury by advanced imaging and other biomarkers along with the ultimate linkage of these markers to specific impaired biological processes. Proteomic and genetic methods have promise to identify more homogeneous subsets of patients that may be targeted for specific therapeutic interventions in the future.
